# Digital feedback during clinical education in the emergency medical services: a qualitative study

**DOI:** 10.1186/s12909-023-04138-y

**Published:** 2023-03-14

**Authors:** Tomas Nilsson, I. Masiello, E. Broberger, V. Lindström

**Affiliations:** 1 Karolinska Institutet SE, Stockholm, 11883 Sweden; 2 Department of Computer Science and Media Technology, Linnaeus University, Växjö, Sweden; 3 Department for Neurobiology, Care Sciences, and Society, Division of Nursing, Karolinska Institutet, Huddinge, Sweden; 4 Department of Nursing, Umeå University, Umeå, Sweden; 5 Department of Ambulance Service, Region Västerbotten, Umeå, Sweden; 6 Department of Health Promotion Science, Sophiahemmet University, Stockholm, Sweden

**Keywords:** Digital Assessment, Formative Assessment, Reflection, Clinical Education, Emergency Medical Services

## Abstract

**Background:**

Clinical education is essential for students’ progress towards becoming registered nurses (RN) in Sweden. Assessment of caring skills in the Emergency Medical Services (EMS) is complex due to the ever-changing scenarios and the fact that multiple supervisors are involved in the student’s education. Currently, assessments of student’s skills are summative and occur twice during the six weeks of clinical education. A digitalized assessment tool (DAT) with an adaptation for formative assessment is a new approach to assessment of nursing skills in the EMS. Since new technologies and changes in procedures are likely to affect both students and supervisors, our aim in this study is to describe students’ and clinical supervisors’ experience of formative assessments using DAT in the EMS.

**Method:**

This study is qualitative, using semi-structured group interviews (*N* = 2) with students and semi-structured individual telephone interviews (*N* = 13) with supervisors. The data was analysed according to Graneheim and Landman’s method for content analysis. This analysis generated 221 codes organized into 10 categories within which three themes were identified. The students in this study were nursing students in their last semester and all supervisors were experienced RNs.

**Results:**

The results showed that students and supervisors had mainly positive views of the DAT and the formative assessment stating that the information they provided while using the DAT offered opportunities for reflection. The DAT supported the students’ learning by visualizing strengths and areas of improvement, as well as displaying progress using a Likert scale. The application improved communication, but additional features linking the assessment tool with the university were requested. The application contributed to transparency in the assessments and was seen as preferable to the traditional ‘pen and paper’ method.

**Conclusion:**

A digital system was described in a positive manner, and the assessment using the DAT facilitated reflection and formative assessment. The use of a Likert scale was considered positive in order to demonstrate progression which with advantage could be demonstrated visually.

## Introduction

Clinical education is an essential part of the training to become an RN. The Emergency medical services (EMS) in the Stockholm area introduced a structured clinical training programme for nursing students in 2012 and it is now an accepted setting for clinical education within the EMS. During clinical training in the EMS, students face a challenging, ever-changing learning environment that tests their clinical reasoning and caring skills [1]. In addition, students face challenges when working with multiple supervisors and inconsistencies in assessment with a summative approach, which does not support their learning on a day-to-day basis [2]. With digitalization, the assessment tools may become accessible in the field, and assessments of caring skills can move in a formative direction that may benefit students’ learning. The formative assessment and documentation of it can be facilitated through daily assessments, creating continuity for students. This study aims to describe students’ and clinical supervisors’ experience of formative assessments using a DAT in the EMS.

## Background

### Clinical education in the EMS

The care provided by the EMS is characterised by short patient encounters, ever-changing environments, and patients seeking interventions for all kinds of complaints [1, 3]. The EMS differs from other clinical education settings because of the lack of time to prepare for patient encounters and the fact that it is difficult to interrupt or step out of the encounter to discuss strategies or engage in pedagogical reflection. Because of this, and the fact that the staffing and organisation of the ambulances differs greatly both nationally and internationally, the clinical education of undergraduate nursing students in the EMS is uncommon. Internationally, staffing in the EMS ranges from physicians to paramedics with several years of training to emergency medical technicians (EMT) with education ranging from weeks to years. In Sweden, at least one member of the EMS crew is an RN, and in some regions the RN also has additional training in emergency care. Despite all this, earlier research has demonstrated that nursing students are able to learn caring skills in the EMS by interviewing, assessing, treating, and evaluating patients on a daily basis [1, 4–6].

### Feedback and assessment

Feedback during clinical education is crucial for developing caring skills and to preparing students for work as RNs [7]. At present, assessment of caring skills happens twice during the student’s clinical education in Stockholm. A university teacher and a clinical supervisor are usually present, and feedback is of a summative nature [8]. Students also receive feedback from their supervisors on a regular basis during their clinical education, and this feedback can be considered formative. Formative assessment, however, can be affected by several factors, such as the clinical setting, the student’s own strengths, workplace challenges, expectations, and the student’s social network, prior knowledge, and skills [9–11]. In addition, a heavy workload and staff shortages in the clinical setting make it difficult for clinical supervisors to find time to assess students properly [12]. Further, the relationship between supervisors and students can affect the assessment in both positive and negative ways [8]. Having multiple supervisors can also affect the student’s progress negatively. Earlier research suggested that additional supervisor meant that students regressed in their progression with every new encounter and can also add to challenges in communication and documentation between supervisors [2, 13]. Nonetheless, the use of formative assessment has been shown to be effective not only for deep learning, but also for increasing motivation and building self-esteem [14].

### Technology in clinical education

Digital technology is nowadays an integral part of health sciences education programs, and one of its uses is for communicating. Communication between students and supervisors is a challenge and the use of digital technology could improve the communication [15]. On the other hand, certain factors may negatively affect communication when technology is introduced. Workflow could be disrupted, for example, and the user’s personal attributes and preferences could have a negative effect on the implementation of new technologies [16]. Reynolds and Eaton showed that when technology is introduced in learning activities, technical support plays an important role. Students have reported ‘technological meltdown’ when digital systems malfunction, leading to the conclusion that having robust systems that are well adapted to a specific learning scenario is crucial, and that support must be available. Additionally, it is important that students fully understand the potential educational value of using a digital system in order to fully embrace the transition [17].

## Materials and methods

### Study design

A qualitative design using semi-structured interviews was used to capture both students’ and supervisors’ perspectives on their experiences with formative assessment using a DAT in the EMS. The interview data was analysed according to Graneheim and Landman’s method for content analysis [18].

### The application and the DAT

In this study, a smartphone was used as the assessment medium. An application was installed in the smartphone and the DAT was uploaded into the application. The application was managed by the students, who gave the supervisors access to it and thereby access to the DAT. The application had a predefined design, and the research group could add Learning objective (LO) but could not alter the design or add functions in any way. The smartphone was offline, and no SIM card was installed. The data was uploaded to the cloud server when the smartphone connected to the Wi-Fi at the ambulance station. The application was designed to be used daily while working in the EMS, and every student had a personal account that was activated using a provided password. In the DAT, the LO were presented one by one, and completion of one LO activated the next, until all (N = 11) were completed. When the assessments were finished, students were asked to submit them, which then made them accessible to the researchers. The assessment procedure could be repeated indefinitely. The LO`s in used in DAT were adapted from the prior assessment tool which were constructed from the course curriculum with LO`s designed to embrace all aspects of clinical caring skills. The DAT was constructed in a way were the LO`s were more distinct and phrased in a way were the LO`s were listed one by one instead of using complex LO´s with several LO`s imbedded in one. Earlier research has shown that complex LOs lead to interpretations and inconsistencies in assessment [19]. To ease implementation and validity of the DAT, students and supervisors were invited to participate in the adaptation of the LO`s. In addition, a pilot study was performed to test both the LO for clinical education and the DAT prior to the study, which resulted in minor changes to the LO and to the information given to the students and supervisors. DAT includes a seven graded Likert scale in order to facilitate for formative assessment. In total, the DAT consisted of 11 LO`S which covered different aspects of the caring situations as for example technical skills were the LO read:

### To what extent was the student familiar with the equipment used in the caring situation?

Other aspects that related to the care provided by the student read:

*To what extent did the student plan and prioritize between caring measures from the patient’s perspective?*

### Setting

The study was conducted in the EMS in Stockholm, Sweden, in 2019. The EMS supervisors were RNs with 1 year of additional university training in emergency care and a least 1 year of employment by the EMS. During a student’s clinical education in the EMS, the supervisor is responsible for pedagogical guidance and assessments according to the LO. EMTs are part of the EMS team but have no supervisory duties or responsibilities. The three ambulance stations where the students were placed for EMS training were in densely populated areas with a high caseload. A higher number of patients is essential in order to provide a good learning environment, as this offers ever-changing scenarios and patient encounters [2]. Clinical education took place at all hours of the day for 32 h per week over a 6-week period.

### Participants

A convenience sample of both nursing students and supervisors was used in the study. For students, convenience correlated with their availability to participate in the study during their clinical education period in the EMS, and for supervisors, convenience related to their availability to participate in telephone interviews.

Students were recruited from a university nursing program in Stockholm that consists of six semesters over three years. The education earns the students 180 credits in the European Credit Transfer System at a bachelor’s level. The students included in this study were in their last semester of their program, which focuses on emergency care. In total, 16 students were invited to participate in the study, and 14 chose to participate. The two students who chose not to participate did so due to a fear that participation would negatively affect their studies.

In total, 13 supervisors were asked to participate in the interviews. The requirements were that they had used the DAT on a regular basis and could participate in a telephone interview.

### Data collection

Two group interviews took place after the students finished their clinical education in the EMS and all grades were finalized. The two groups consisted of seven students each. The group interview format was chosen because it offered the students the opportunity to discuss their experiences using the DAT with each other, aiding recall and thus generating richer data. Communication in group interviews differs from individual interviews, providing a greater variety of information and a space where students can use, for instance, anecdotes and humour in the discussion. The researcher’s role becomes passive during group interviews, which in this case is seen as advantageous [20]. The group interviews where a constant flow of discussions which went back and forward between the students with little involvement from the researchers. The interviews were performed by the first, the third and the fourth authors together with a research colleague from the institution. Two researchers were present at each group interview. The first author was known to the participants as a pedagogical support for the students and supervisors as well as a facilitator for university collaborations in the EMS. The others had no prior or current connections with the students. A semi-structured interview guide was used to enhance the discussions. The interview guide was divided into three sections. The introduction focused on a short description of the aim of the study and a confirmation that all participants were aware that participation was voluntary. The students were also informed that the audio from the group interview would be recorded. The body of the guide focused on the experience of digital formative assessment from different perspectives. It started with an open question—‘What was your experience of using the DAT?’—that was followed up with probing questions about the discussion generated by the students. One researcher had the role of a moderator, while the other took notes and provided support when needed to ensure that all participants had the opportunity to speak. The summary section provided the participants with the opportunity to comment on previously undiscussed topics. The two interviews lasted approximately 40 and 60 min.

Individual telephone interviews were conducted with the supervisors. This method was chosen due to their scattered locations and irregular schedules. The interviews were recorded using a smartphone without a SIM card, and a semi-structured interview guide similar to that used in the group interviews with the students was employed. An introductory section explained the research, its aims, and the voluntary participation. The main section focused on learning and assessment, collaboration with students, and further development of the DAT and LO. The final section provided the supervisors with an opportunity to comment on previously undiscussed topics. Probing questions were asked as the interviews progressed. The interviews lasted from 8 to 26 min, with an average of approximately 17 min.

Both the group and individual interviews were transcribed after completion of the final interview.

### Data analysis

Data analysis was performed using a manifest, inductive content analysis according to Graneheim and Lundman [21] [18]. Initially, the transcribed material was read several times to create familiarity with the data. Next, the data was broken down into meaning units containing sentences relevant to the study (*n* = 221), which were organized electronically using Microsoft Excel. These meaning units were then further shortened into condensed units, meaning that the sentences were stripped of unnecessary words, preserving the core content. During both steps, this condensation was performed with as little abstraction and interpretation as possible to preserve the participants’ own phrasing. The condensed units were then further shortened into codes consisting of single words or short phrases, and then organized into sub-categories (*n* = 20) where similar topics were discussed. Sub-categories were organized by colour to easily distinguish patterns, resulting in the construction of categories (*n* = 10). The last step of the analysis was to thematize the data (*n* = 3) by identifying common traits within the categories.

The analysis was initiated by the first author and the condensation in each step was discussed with the last author. The analysis moved back and forth from codes to sub-categories and categories until a credible pattern could be distinguished. Finally, the other authors were presented with the finished analysis and data, and the analysis was discussed in a workshop. These measures were taken to ensure rigor and minimize bias in the analysis. The result is presented in a sectional matter in order to highlight the different perspectives that students and supervisors had in regard to the DAT.

## Results

The three themes extracted from the data were *formative assessment using DAT, multiple supervisors* and *digital assessment tool*, and each theme contained two categories.

### Formative assessment using the DAT

#### Assessment

##### Students

Students believed supervisors should be responsible in the assessments done using the DAT. They also said that patient encounters influenced the usage of DAT and the generated assessments: more complex and time-sensitive calls could result in negative feedback, since students were unable to perform necessary tasks or draw relevant conclusions, in contrast, encounters with patients with little or no obvious need for medical attention produced scant feedback. One student noted in this respect: ‘It will vary every day depending on what patients we encounter’. Students preferred the LO´s in the DAT to the original, although some LO`s lacked relevance in the given context. In this regard, students commented, ‘The learning objectives in the application were more concrete and concise’ and ‘Some learning objectives were hard to apply’. The students discussed the social aspect of assessment using DAT—saying, for example, ‘personal chemistry affects the assessment’—but made conflicting comments as to whether the assessments affected the collaboration between students and supervisors.

##### Supervisors

Supervisors emphasised that DAT was useful when they planed their pedagogical strategies since it created opportunities for reflection with the students, with one saying, ‘It highlighted areas of improvement’. They also remarked that for the assessment to be of any pedagogical interest, it needed to be completed jointly with the student, and that the given grades needed to be discussed together. The DAT was considered advantageous due to the fact that it was always close at hand.

Supervisors had conflicting opinions about the LO`s used in the DAT—saying both, ‘The learning objectives in the application were better’ and ‘I preferred the old learning objectives’—but they also expressed a need for clarification of the grading scale with clear, relevant examples. Supervisors noted that the frequent assessment with the DAT positively affected their interactions with students, saying, ‘It was very easy to use. If anything, the app contributed to a better relationship with the student’. Supervisors said that in order to maintain a high frequency of assessments the usage of the DAT needed to be mandatory, and that frequency was important in providing a good overview of the students’ progress.

##### Students and supervisors

While both students and supervisors described the grading scale in the DAT as positive because it highlighted progress. They also struggled to differentiate between the grades in the Likert scale. This illustrated a need for clear examples and support for both groups. Both students and supervisors remarked on the lack of clarity in terms of the knowledge each LO represented, as well as the expected performance levels of the students, saying, ‘The supervisors struggled with understanding the grading steps’ and ‘the steps were hard to understand’. Students and supervisors also said that using the DAT too frequently could be negative and that every assessment needs to be concrete and bring up something new so that the student can learn from the feedback: ‘One of the dangers is that it becomes arbitrary’.

#### Feedback

##### Students

Students said that the DAT was a good pedagogical tool for supervisors with less experience who were insecure in their roles. It gave these supervisors structure and helped create a comfortable situation for giving feedback: ‘It was a good support for supervisors who were a bit insecure’.

##### Supervisors

The supervisors remarked that the DAT ‘created possibilities for reflection’ and that the LO were discussed more frequently. They further said that the use of the DAT highlighted progress and was useful for guiding the students.

##### Students and supervisors

The students and the supervisors agreed that the DAT was advantageous and created a positive pedagogical climate, saying, ‘it encouraged daily feedback’ and ‘every patient encounter was discussed’. Both students and supervisors agreed that discussion and reflection were important parts of the learning process, and that formative assessment was preferable to the standard summative feedback. Both groups saw the DAT as a facilitator for this in contrast to prior assessment tool. Both groups also said that feedback was a challenge, but the DAT was helpful: ‘It was an aid when communication was lacking’. Both groups further claimed that assessment with the digital tool caused them to reflect on daily activities and that it was a good way to assess the student’s progress, saying, ‘It highlighted what needed to be focused on’, and ‘it was a good way to summarize the day’.

### Multiple supervisors

#### Supervisorship

##### Students

Students recognized that having multiple supervisors had advantages and disadvantages, saying, ‘I wished I had more supervisors’, or ‘Having multiple supervisors was negative for my progression’.

Having different perspectives on the care provided and different pedagogical strategies were some of the benefits cited, while a lack of continuity that caused difficulties for the students was described as a disadvantage. The DAT was considered positive in both regards because it could capture the different perspectives in that the lack of continuity could be less tangible when assessments became accessible in the field.

Students said that the supervisors’ experience of working in the ambulance affected the assessments, saying, ‘it made a big difference if they had been in the business a long time”, and noting that different supervisors offered different perspectives. The DAT was useful in order to bring these perspectives into the light through reflection and discussions.

##### Students and supervisors

Both students and supervisors recognized that having multiple supervisors influenced students’ learning. Students and supervisors alike concluded that assessment varied between supervisors, saying, ‘everyone assesses differently’, and ‘different supervisors make different assessments’. Different explanations were given for this conclusion, such as the supervisor’s personality, education, motivation, and experience in the EMS. The DAT ability to document the assessments were seen as an advantage where students became less dependent on one single supervisor’s assessments.

#### Support

##### Students

Students stated that the DAT was helpful when multiple supervisors were engaged in their education because it created a common foundation for discussion, saying, ‘It was good to have the support when many supervisors were involved’.

##### Supervisors

The supervisors said that the DAT could serve as a hub for supervisors who are not frequently involved in clinical education, saying, ‘The application can function as a reporting system between supervisors if there are many involved’ They also noted that the DAT could be used for communication between supervisors, remarking, ‘The application can be good for communication between supervisors when many are involved’. The supervisors also highlighted the need for learning from other supervisors by accessing other supervisors’ assessments. Reading earlier feedback gave the supervisors an opportunity to reflect on their own pedagogical strategies: ‘it was good to see other supervisors’ assessments’.

### Digital assessment tool

#### User-friendliness

##### Students

The students criticised the user-friendliness of the application, saying, ‘it was complicated’, ‘goofy’, and ‘hard to access in the beginning’. Students blamed this on poor preparation and a lack of information about the application and the assessment instrument.

##### Supervisors

The supervisors reported that the assessment tool was easy to use and was very clear. They stated that due to the pressured work situation, with little time between calls, it was hard to reflect on the students, saying, ‘It was hard to find the time to use the application’, and ‘There should have been time set aside for the assessments’.

##### Students and supervisors

Both positive and negative aspects of the DAT were mentioned. Students and supervisors stated that it was quick to use, saying, ‘For me it took a few minutes’, and ‘the assessments took a short amount of time’.

#### Technical aspects

##### Supervisors

The supervisors emphasised that the accessibility was a plus: ‘The greatest strength was that it was always there and that it was effective’. They also claimed that the smartphone was preferable to the traditional ‘pen and paper’ tools. Furthermore, the supervisors saw the possibility for the application to be a communication tool for all supervisors, where feedback could be gathered and accessible to both students and supervisors, saying, ‘The application could be managed by the students and serve as a hub for us supervisors who supervise irregularly’.

## Discussion

Overall, the impression was that formative assessment using a digital tool in the EMS felt modern and innovative.

### Formative assessment

Participants claimed that the DAT supported reflection and facilitated formative assessment, which was considered preferable to summative feedback. This notion is supported by earlier research [22]. The DAT also offered the possibility to mix summative and formative assessments and feedback. These mixed feedback methods have previously been discussed by Watling and Ginsburg and are generally seen as positive, although they must be handled carefully in order to be effective. Supervisors and students alike need clarity about the difference between feedback and assessment and which is used when [23].

The grading scale was a challenge as well as a facilitator for formative assessment in the sense that it displayed progression, but it also generated discussion about the definition of requirements for each grading step. This is seconded by earlier research that shows both strengths and weaknesses with pass/fail grades as well as with grading scales with multiple steps. Students subjected to pass/fail grades has been showed to produces similar academical results as students assessed with five-step grading scales. Other research supports arguments that multiple-step scales offers the opportunity to measure students’ academic performance and hence guide the students towards improved performances, which is the intended effect of formative assessment [24].

### Multiple supervisors

Receiving assessments from single versus multiple supervisors was discussed as being both positive and negative. Earlier research supports the use of single supervisors [2], but both students and supervisors were able to see the benefits of multiple supervisors in combination with formative assessments. Multiple supervisors offer a wider perspective on the care provided by the EMS personnel due to their different backgrounds and experience, both as supervisors and clinicians. The difference in the assessments can be seen as a result of the difference in background and experience but by documenting the assessments, objectivity can be reached through reflection on the variations.

### Digital assessment tool

The DAT made it possible for students and supervisors to perform continuous assessment that was documented and accessible to the researchers. The application was seen as a positive measure because it collected and documented all assessments and could be used as the basis for summative assessments, thereby validating the grading process.

In their 2019 paper, McInerney and Druva discussed the importance of clinicians’ attitudes towards the development and implementation of new systems. These attitudes are believed to have greatly impacted the data in this research paper. Progress-minded supervisors saw benefits and few obstacles, saying that assessments were fast and easy, while others claimed that it was hard to find the time. The same could be argued about the students, with some more negative than others. This negative attitude, it should be noted, may have been caused by a lack of information and technical support.

The application we used was not specifically designed for this kind of assessment, making it a bit too rigid to be fully effective. Using a more flexible application that could be adapted to the intended DAT and had the ability to add additional features and communication channels would probably have been preferable, a claim that is supported in prior research [25]. Transparency in assessments was discussed primarily by the supervisors, who saw that the application could serve as a hub where they could access other supervisors’ prior assessments—something that could be helpful in formulating their own assessments but also be a source of learning. If notes could be added to the assessments, supervisors could learn from each other and develop their own pedagogical reasoning, and for the students, notes could clarify their need for further training or serve as documentation of their progress.

## Conclusion

Students and supervisors described that using the DAT stimulated reflection and improved communication to a greater extend then when LO`s were assessed in analogue format. The digital system used in this study was described as modern and progressive, and, with some improvements, the continuous assessments can facilitate reflection. The Likert scale is useful to describe progress, but the grading scale needs to be clarified in order to be efficiently used. Formative assessment is considered preferable to summative feedback that usually dominates clinical education, and the DAT serves both as a generator for reflection and as a reminder for students and supervisors to make time for reflection. The experiences described by both students and supervisors indicates that making the transition to digitalized formative assessment is beneficial from a pedagogical as well as an administrative standpoint. The application needs to be adapted to its unique purpose and equipped with functions that summarize the assessments and displays them visually. In order to bridge the gap between the clinic and the university, the DAT can serve as a communication channel so that the university can monitor the students’ progress in real time, which in our study is considered an advantage. When change is implemented in the organisation clear instructions and support is necessary to ease frustration and handle unforeseen events.

## Limitations

There are several limitations to this study, the primary being that two members of the research group are well known in the EMS in Stockholm. This could have influenced the supervisors’ answers during the interviews. The data however contains both positive and negative experiences of using DAT which suggests that the bias was limited.

The second limitation relates to the students’ clinical education in the EMS. Prior experience has shown that the EMS is a demanding placement for students where quick diagnostics and caring is needed, and patients have many different complaints [2]. This certainly limits the external validity of this study, where reproducibility is weakened. In addition, adding a new DAT may have caused the students additional stress, thus influencing their views of the study. This could be seen in a variation on how frequently the students had been using the DAT. However, a less stressful clinical education situations could have a more positive result in relation to DAT. An environment that offers possibility to dedicate time and a fix, quite place for assessment and feedback would probably improve the quality and thereby a more positive view of the DAT.

The third limitation lies in the data collection, where group interviews could be a limitation. Some individuals may have had an impact on the other students, steering the interview in a certain direction instead of creating an open climate for discussion. Prior studies have shown that group interviews may generate more criticism than individual interviews [26], which is evident in the data gathered in this study. Thus, it could be argued that performing individual interviews with each participant could have been advantageous. The data, however, were coherent between the two groups, suggesting that this effect was minor.

## Data Availability

The dataset generated and analysed in this study is available from the corresponding author on reasonable request.
